# Supplementary figures and images for: A study of repetitive sequences in the genome of Sinopodisma qinlingensis
Source: PeerJ. 2025 Apr 30;13:e19358. doi: 10.7717/peerj.19358 (PMC12049104; doi:10.7717/peerj.19358)

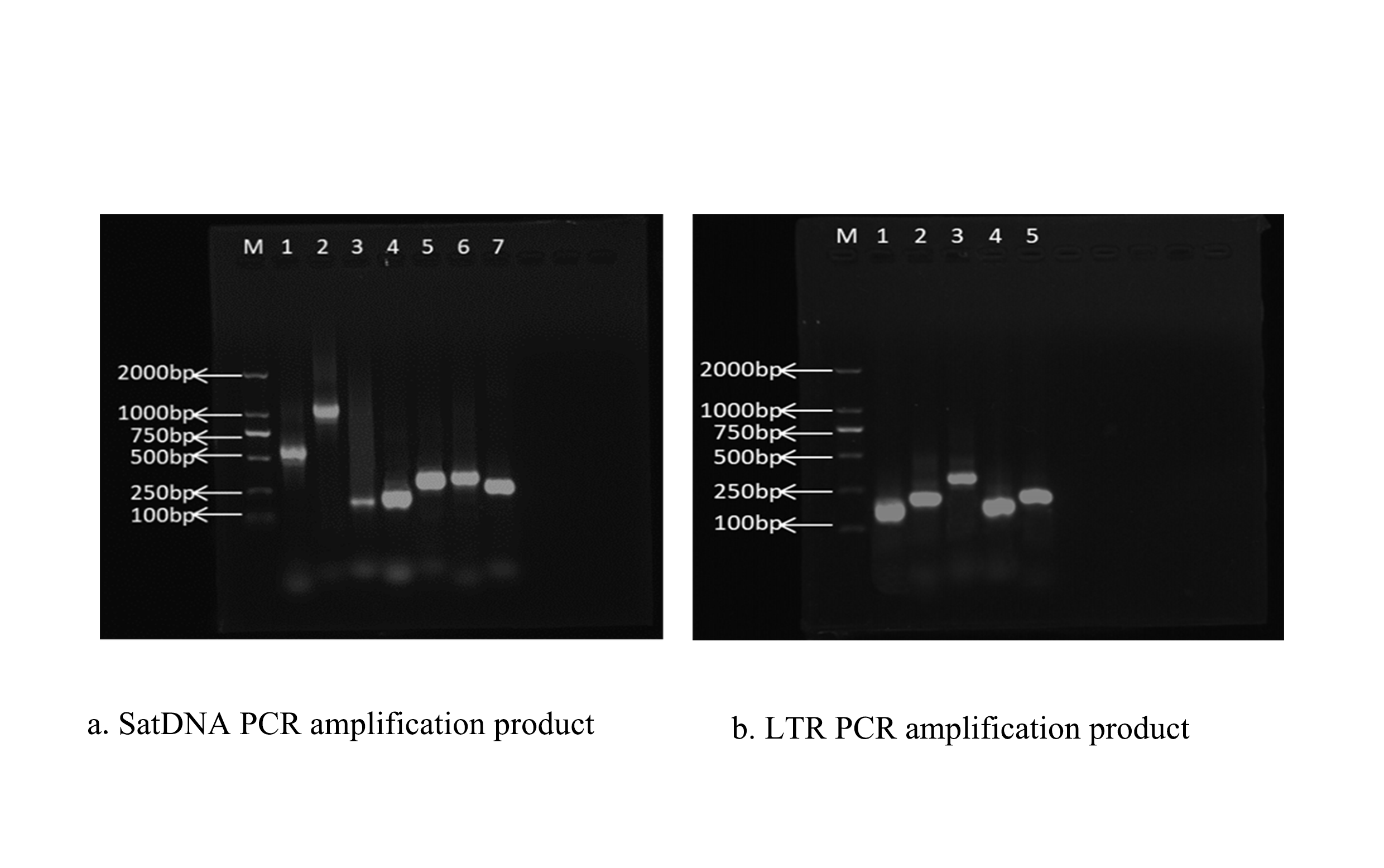

Supplement: Supplemental Information 1 [file peerj-13-19358-s001.png]

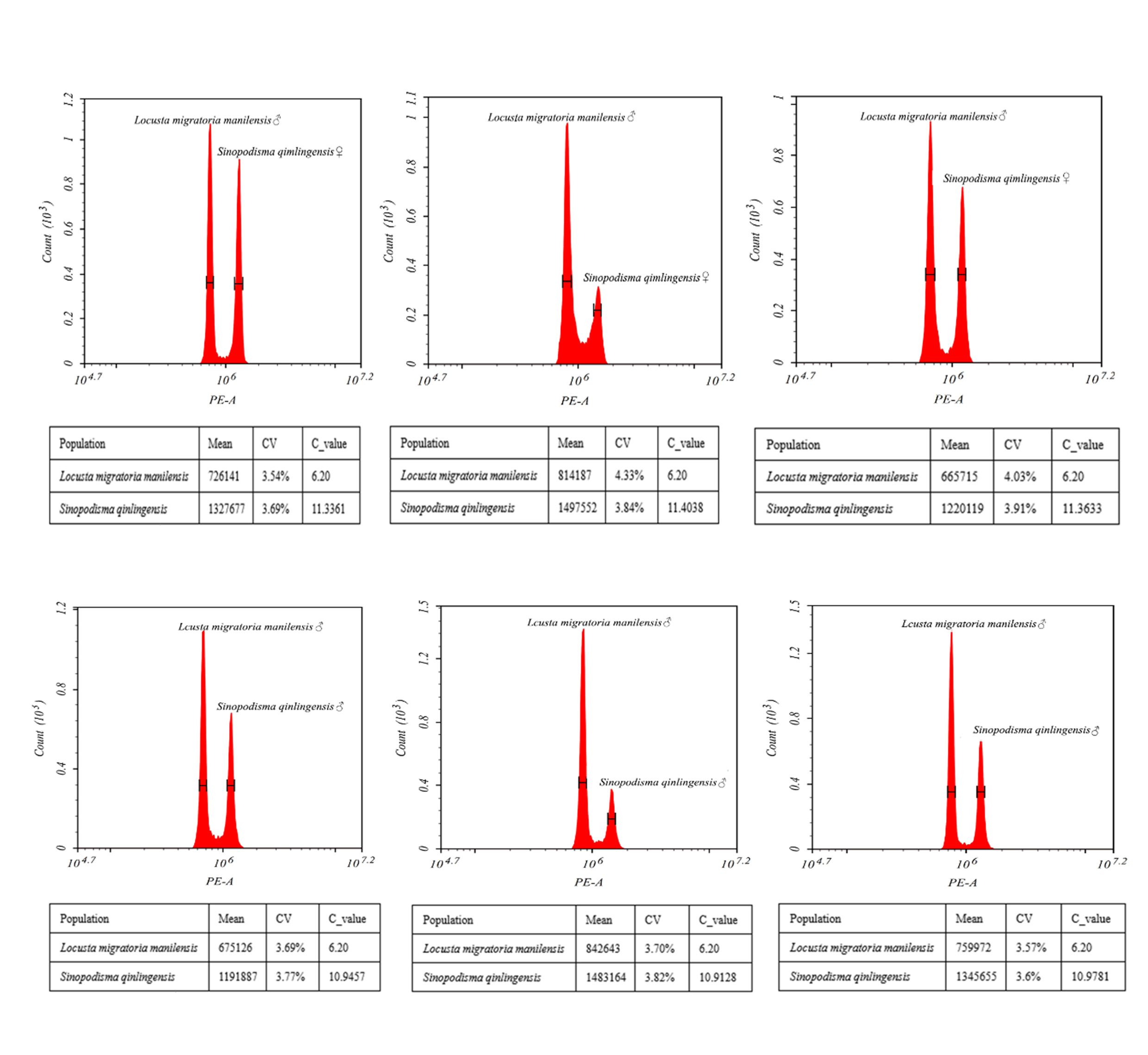

Supplement: Supplemental Information 2 [file peerj-13-19358-s002.jpg]

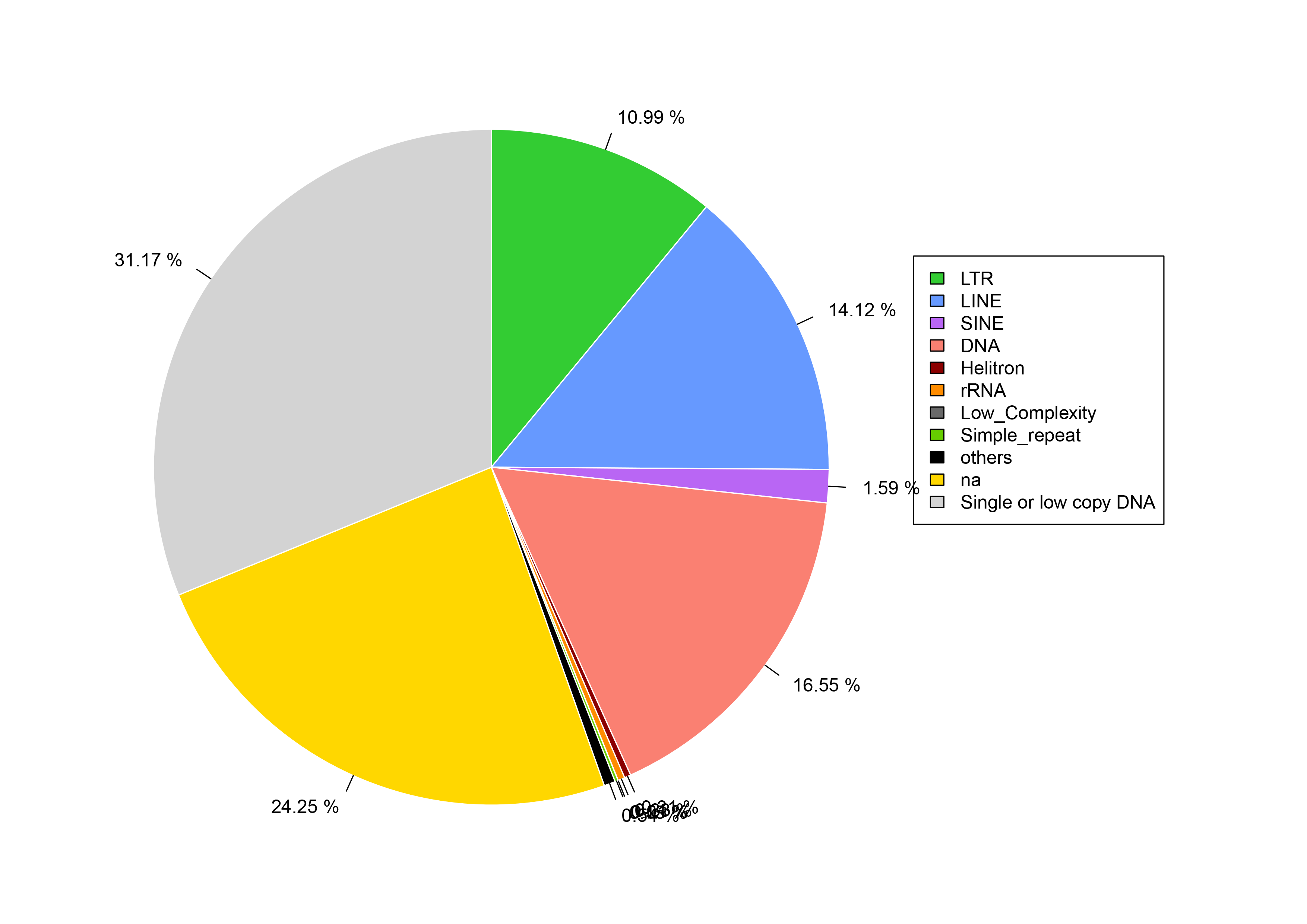

Supplement: Supplemental Information 3 [file peerj-13-19358-s003.png]

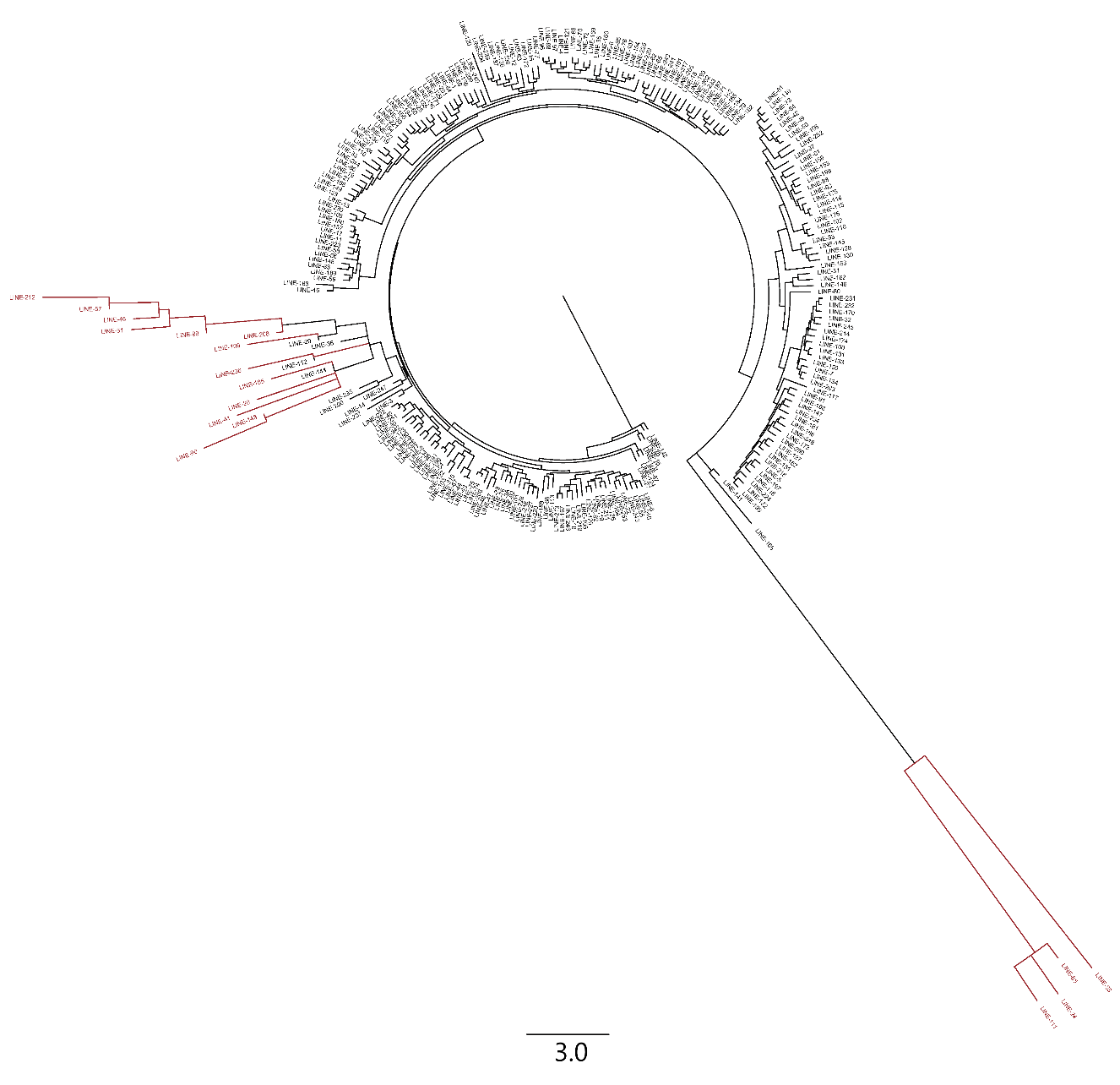

Supplement: Supplemental Information 4 [file peerj-13-19358-s004.png]
