# Supplementary material for: A study of repetitive sequences in the genome of Sinopodisma qinlingensis
Source: PeerJ. 2025 Apr 30;13:e19358. doi: 10.7717/peerj.19358 (PMC12049104; doi:10.7717/peerj.19358)
Supplement: Supplemental Information 5 [file peerj-13-19358-s005.docx]

Table S1 The primers of 5 satellite DNAs and 5 LTRs and annealing temperature of PCR

| Name | Upstream Primers (5’-3’) | Downstream Primers (5’-3’) | | Anneal Temperatur(℃) |
| --- | --- | --- | --- | --- |
| SatDNA-01 | TCTTAGTTACCTGTTCCCAAAT | | CCGAGAAGCGATACCATAC | 56 |
| SatDNA-02 | TCGCATCAACCAAATCTC | TGTACCGTTAGTGGAAAGC | | 54 |
|  |  |  | |  |
| SatDNA-03 | TCACTGTGGTTGTTGTAT | ATTCTAAGGGGAGTTGTT | | 48 |
| SatDNA-04 | GCATCGCATATCGACCAGGCACT | CCATTACACCACGGAACCAGACG | | 56 |
| SatDNA-05 | GATACCTGGCTTTCACCC | CACAACGCAATGTCCTAAC | | 54 |
| SatDNA-06 | GACCACTAAGCCAAGCAA | TAGGCACTATTCCGCATC | | 53 |
| SatDNA-07 | GAATCCGAATACAATAGCG | TCCTGGCATAAGAATCAC | | 52 |
| LTR-01 | GCGTAGCGGCAGTGCTTTCTCAT | CGTTAAAGGCTTGTGGTCGGTGA | | 55 |
| LTR-02 | ATAAACTGCGGGAACGAC | AGAAGACTGGCAAACTGA | | 53 |
| LTR-03 | ATAAACTGCGGGAACGAC | TCTCCTGGCTGCGTATGA | | 55 |
| LTR-04 | GCGTAGCGGCAGTGCTTTCTCAT | CGTTAAAGGCTTGTGGTCGGTGA | | 55 |
| LTR-05 | CTTCTTACCCACTCCCACG | ACGGAACAGACGCAATCA | | 52 |
