# Supplementary material for: A study of repetitive sequences in the genome of Sinopodisma qinlingensis
Source: PeerJ. 2025 Apr 30;13:e19358. doi: 10.7717/peerj.19358 (PMC12049104; doi:10.7717/peerj.19358)
Supplement: Supplemental Information 6 [file peerj-13-19358-s006.docx]

Table S2 PCR reaction system

| Components | Consumption(μL) |
| --- | --- |
| DNA(>100ng/μL) | 1.0 |
| Upstream Primers (100μM) | 1.0 |
| Downstream Primers (100μM) | 1.0 |
| Biotin -11-dUTP(1Mm) | 1.0 |
| 2×Taq PCR StarMix with Loading Dye | 10.0 |
| ddH_2_O | 6.0 |
| total | 20.0 |
