# Supplementary material for: A study of repetitive sequences in the genome of Sinopodisma qinlingensis
Source: PeerJ. 2025 Apr 30;13:e19358. doi: 10.7717/peerj.19358 (PMC12049104; doi:10.7717/peerj.19358)
Supplement: Supplemental Information 7 [file peerj-13-19358-s007.docx]

Table S3 Flow cytometry to determine the genome size of *S.qinlingensis*

| Gender | Genome size | | Average value | Standard deviation |
| --- | --- | --- | --- | --- |
| female | 1 | 11.3361pg | 11.3677pg | 0.0198 |
|  | 2 | 11.4038pg |  |  |
|  | 3 | 11.3633pg |  |  |
| male | 1 | 10.9457pg | 10.9455pg | 0.0188 |
|  | 2 | 10.9128pg |  |  |
|  |  |  |  |  |
|  | 3 | 10.9781pg |  |  |
