# Supplementary material for: A study of repetitive sequences in the genome of Sinopodisma qinlingensis
Source: PeerJ. 2025 Apr 30;13:e19358. doi: 10.7717/peerj.19358 (PMC12049104; doi:10.7717/peerj.19358)
Supplement: Supplemental Information 8 [file peerj-13-19358-s008.docx]

Sat-01

| Upstream Primers (5’-3’) | Downstream Primers (5’-3’) |
| --- | --- |
| TCTTAGTTACCTGTTCCCAAAT | CCGAGAAGCGATACCATAC |

序列

GTGTATTATGTGGGCGACGAAAAGTAAAGTCTGTTTATTTTCTTTCTCTCGTTTGCAGTGTGCACTCTTAGTTACCTGTT

CCCAAATTCTATATTTTCTCGCGCAGTCGCATTACGCGTTGCAGGGTACTTCTGGAGAGCCTTCCCAGCGTTTTGGCGGT

GCCTGTGGGCGGCTCTCAGGTGCATGGGGCGACCGTACATATTTTAATTTTTGGCAGAGATCTCGTCTTTTCGCGAGCGT

CGTCGTCGCCGCCGCACGCACGCAGTACACATGTGTGCAGATACGCACAGCCATTCAGATGGTGAAGAGTAAACTGTATA

GGAAGCTGTAGGACGGCGGTGTAAGCTCGCGTGTCACTCGCGCCGGCCGGGCGTAACTCGCGCCGGCCTGCCGCGGCTGT

CACTGGCGCTGGGCAGAGTTCCCTTTCGAAATGCAAAGCACAGTCACACAGGCGACCCCTGCTCCGTAATTGGCGTTCTT

CTCGCCGCCCCTGCTCTTCCAATGTGTTTCAATTTGTCGCCTCGCGTCGGCACTTTGAATCACCTTGTGTCGTTCTCAGA

GGTACAGGGCATACACATGGGGCTTATAGGAGGCGCCATGGGGCCCGTATGGTATCGCTTCTCGGCCTTTTGGCTAAGAT

CAAAGTGTAGTATCTGTTCTTATCAGCTTAATATCTGATACGTCCTGCATCGCAGGACCAGAATATTAAACTCATTTTTG

GCTCTTGACGGAGTGCTAGGGGCTTGCTCCACCTCTGTCGCGGGTTGGCCCGGCATTGCAGTACCGCCGGGATCGGCCCA

CCTAAAAACAATTCAAACAAAGCTGGAAATGAAATTAATAATTCTGCAGTGATCGGATATTATGCTGTTGTCAAAACATG

TTTTGTTTTCCTATTCCCTTGCT

Sat-02

| Upstream Primers (5’-3’) | Downstream Primers (5’-3’) |
| --- | --- |
| TCGCATCAACCAAATCTC | TGTACCGTTAGTGGAAAGC |

AATACCTGTAGAAAACACGTGCGCCTTACTCACCGCTACCTGTCCCACAGAGAGTGCATGTGTGTGGCGGACTCTAAGCT

CTGCAGGTGCACGGCGGATGGCCAGCTGGACGGGACGAGAGGTGTCGTCCTCGGGAACTGGAAGTCGCCCCAGCCCACGG

AGGGCCGGAGTGACCACACCGCCGATGCCAGAGGGTCGCATCAACCAAATCTCCTTCATTTACCACTTGAACTTCCCTGG

TAGGTCCACAAGCAGGCAGTGGCCCTGTGGCGCAATGGATAACGCGTCTGACTACGGATCAGAAGATTCCAGGTTCGAAT

CCTGGCAGGGTCGTCATTTTGCTAGGTGGAGGTAAGTACTTAGGCAACAGTTGTCGTGCAGGTCAGTGTTACTCGTGCTG

TGTCTCGCAGTTGCTTGCCGAGTCCATTCGCGGTAAATACGGCCGCCCACAAGCGTCAGCGTAGCGTCAGCTGAGCTAAG

TTGAGCCAAGTCGAGCCGACACAAGGTGTTTCTCAATTACAATCAGGCGTATGACAGCAACTCAAACAACACCCTCCATG

TAGCATGCTCGAGGGGCGACGTTCGAAAGTCTAGCAGAGTGGCGCAGTGGAAGCGTGCTGGGCCCATAACCCAGAGGTCC

GTGGATCGAAACCACGCTCTGCTAAAATTTATTTGGTCTCCTATTACTTCGAGACCAATTATTAAGCCGAAAAGAAACCG

CTTACTGCTAGCTCAGCACTGCAAAAGGTTAGTCTGTAATTCTGAAATGACCTCATCGTGCAGAAGGGAATTCGAAAAGT

CACTTTCGAAGCTGAAGTAAGTTTTGTACCATTTTCACTCCTGGGTCAGAATTTCATTTTTCTTCTGGCCCAGACCAGCA

GCATTTGCGCAGTCTTACAGTGGTAAAATCGCATCTCCCCGGCGGGGAATCGAACCCCGGTCTCCCGCGTGACAGGCGGG

GATACTGACCACTATACTACCGAGGAACACGCCGCATGTGTCACGAATACTCTCTACTGTTTGTAAAGGGGGTGTTTCCT

CTCAAACCTGTCATCCGGAGGCCATCAGAGGTTTGCTGCCACAAAATAAAACTATGCCCCAGGTGAGGCTCGAACTCACA

ACCCCGGCATTGCTCACGGCTACTGCCTTATAAGTACCGTGCGCTAACCAATTGCGCCACTGGGGCTACGTCCCGCTGCT

TTCCACTAACGGTACACTACTCACTTTAAGGCAAGTGCTTACTGCCAGGAACACTGCATGTCATCCTCACCTATGGCCGT

ACTGCAACTTTAGTTAACTGCCTACGAATGCTTAGTACTATTCCCGTTTCATTGCCTCAGCTCATTTGCAAATCGGCCGC

TTCTCTTCAACG

Sat-03

| Upstream Primers (5’-3’) | Downstream Primers (5’-3’) |
| --- | --- |
| TCACTGTGGTTGTTGTAT | ATTCTAAGGGGAGTTGTT |

TGTCACTGTGGTTGTTGTATTAGAGACCAATTTGCAATTATCTCATGCAACTGGGATGTTGATTGAAACTGCTCCTCACG

GACATCTTCCCTGATGAAGCAGTACAACATCGACATACTATAGCGGCTGCAAGGCAGAGTGATGAGCCTCGGAAGGAAGC

AAGAGGTATTCACCTCCTACTAACCATCTCATCCATATAAAAGCAACAACTCCCCTTAGAATAAATTCAATTTGTTAAAT

AATAGATAATTATCTATATAGTTTGTTCACGTT

Sat-04

| Upstream Primers (5’-3’) | Downstream Primers (5’-3’) |
| --- | --- |
| GCATCGCATATCGACCAGGCACT | CCATTACACCACGGAACCAGACG |

CGTAAAGGCTCTTCATCTTGCAGTTGAAGACCCACCCTTTCGGTATTAATCCTAGTGGCAAAAGCCATATCTTCGACAGC

AATGCCGATTCTTGTGCGGACTGCACTTCATATTTTGCATTGCATGGTCCCACCTTGAACTCGGTGTATACCTTTCACCA

AAACTCCGCGCCACAGTCGCGAAAACGATCCCGAACGATTTCAGGCAGGTTCCACCGAGATTCGAACTCGGATCGCTGGA

TTCAAAGTCCAGAGTGCTAACCATTACACCACGGAACCAGACGTGCTTGCCATGTCCTCATGCCTCCTCTTCAAGTCATG

CAGCCAGGCTCGCTTGATATGAAATGAGTTACGGACGCAGATTACTGTCGAAAAGTTTCATCGAATGACGATATTTTCAC

TGTCCTGGGATGAAATGATATCACAGAGGTGAAGCAGATCTTTGAGAATCCGAATACAATAGCGAGAGAGTAGCTACTGT

GGTAGGTCCAGGCTTAGGAAAGCTGTTAAAGTGCCTGGTCGATATGCGATGCTGCAGTTAGTAACTGTGGCAAGATCTCT

TCGAGTTGCGAAGCGCACAGAGCAAGTTGTGTGCGGCCTGTTGGTCTAGGGGTATGATTCCTGCTTTGGGTGCAGGAGGT

CCCGGGTTCAAATCCCGGACAGGCCCTTCTTTTACCTGACGCGTCGTGGGTCTCTAATACTGTTGTAGGTAGGAAGATGA

AACCTCTGTTCAGGAGTCTAGCATGTGTGAGTTATGTGAAACGTGATTCTTATGCCAGGATATAGCTCTCTTAGTTTACT

TCAATGTAGTCAAGAAAACCGGCAACTGAGATGATTCTCCACGTTGCTCTGAAGCTAGTGTATAGCTCACATAGACTCGT

CACATGCCTCATTATGTAGCACCGAAAAAGGGTAAGATGTGGTAT

Sat-05

| Upstream Primers (5’-3’) | Downstream Primers (5’-3’) |
| --- | --- |
| GATACCTGGCTTTCACCC | CACAACGCAATGTCCTAAC |

AGGGACGTCGAACTGTACCGTTCTTTCACGACTGAAAGTAGGCGCTGTTTGGTCAGGTGAGCAATTGCGTAATTACAGAT
GTCCTGCACATGACACATCGAAAAGCGAATGATTGCTACTGTCCAGAGCACGGAAGTTTCGTATCTTGGGGAAAGAACAT
TTGGCCTTCCGGTGTGGTCTAGTGGCTAGGATACCTGGCTTTCACCCAGGAGGCTCGGGTTCGATTCCCGGCACCGGAAT
CGCACATTTTTGTCGCTCCCTATATGCGACTTGTCTGGACTTTGCTCGGCTGACGCTCCGCTGACGCTTGCAGAAGGTAC
CCTAAGTATCGTTTCATGATAACAACTGACGGCGAGAGAGATGCGAAATGTCATTTAAACACGAGATGTCGACCGAGACT
TCACGGCACTTTGTGCGACACTGAGAGCCGGCCGTTCCTGTGTGTCGACACTTGCAGCCTCAAGTGGGCTTGCGGCGGCT
GTCGCCATGGCCGTGATCGTCTAGTGGTTAGGACATTGCGTTGTGGCCGCAATAACCCAGGTTCGAATCCTGGTCACGGC
AATTTTGAAATCATTTTGCTTTGCTGCCACGGCAGCTTGTGACTATGTACTAATGTTGAAATCACAGCACTCCCTTCAGT
TTCATTCACGTTCTGATGGGCGCTGTTCGCCCTATAGTTTCGACGCCGACCGTTATTGGCGCCTCCCAGAACGCAGAAAG
TTGATTTAGATTTTAATCTGTAGTCGAAAGAGGCAGTTGTTGGAGAAGAGATGGATGAGCAGCTAATCGCAGCAGGAATG
TTCTCGGCTGTTCGCGTTCACTGTTCGCAAAAGCTGCGATCAGATACGCACAACCAGTCTGCAGGTAGCGTGGCCGAGCG
GTCTAAGGCGCTGGTTTAAGGCACCAGTCTCTTCGGAGGCGTGGGTTCGAATCCCACCGCTGCCACGTGCGTGAGTTTTT
CCCTCTCGAAGATAGTCGTCGTCAGCACCTGCTTAGGGAAGAGTGCAACCGAGTGGGAGTCCAAGTTTTCTTGCGATCTG
GGTTGGTGCAACGAGACGACGGTGGGACACCTGTCAATAGCTTGAAACGAACGCAGAGAAAAGTGTCACGGGAGTGGCGT
GGTACCCAAGTCGGCGAGGCAGTGACG

LTR-01-5444

| Upstream Primers (5’-3’) | Downstream Primers (5’-3’) |
| --- | --- |
| GCGTAGCGGCAGTGCTTTCTCAT | CGTTAAAGGCTTGTGGTCGGTGA |

ATAAACATGGTTGAGGCCGCAACGCCTGAGCACTTGCTCACGACTTTTCTTCAGTTTCAGAGTGAGCAATTCCGCAAGAT
GCAGGAGGCCCAAAGCCAGCAACTCGCCTCTCTGCAGAACACGTTTTTAGAGGCCCTCCGCGCCCTAGAAACGAGACAAA
CTGCTACCACTCCTGCCGCCCCCACACCTGCAGTGCCGCCCTTCCGTCAGTACGACGCCGAGAACGAGGAATGGAACGAC
TACCGGATGCAGCTTGAGCAGCACTTCACGGCGTACCGGATCACAGGTGAATCGCGCCGCTGCTTTTTGCTTGCATGGTT
AGGTCCCTCGTCATTACAAATTTTGAAGAAACTCTTCCCCGATAAGTCCCCAGATGCGTGTGACTACAGTGTACTTATTC
AGGCATTAGACAAATACTTTGACAGTCAAGTGCATGTTACTGCGGCACGATACAAATTTTTTCGGCTTAAAAAGAGGCAG
GATCAGTCATATCGTGAGTGGTTAGCAGATTTAAAGGGCATGACAAGACATTGTAAATTTGAATGTGAACATGGTGGTTC
CTTTGCCGACCAAATGATTCGTGACTCTATAATTCAAAATGTGCAGGACTCACGAATTCGCTCAAAATTGCTCAGGCTCC
CTAATCCTACATTGCAGGAAGTGTGTGACATAATTGAGGGACAAGAAACTTATTATCACGCAGAGACAGATATGTACAAT
TCATGTCCGCAAATTTCGCGAGTAAGCACTCATAGCCAAGCCACACGGAAAGGTAAACATGCTCGCGGCCGTACAGGTCA
ACGCGGTACTCAGGCCCGCGGTAAACAGTCACGCGGCACCGCCCCGGCTCAGCATGTAAATTCAAATTCTGGCAAGAACT
TTCACTCAACATTTCGTTCCACGAACAATTCTTTAAAGTCTTGCCCCCAGTGTTTTGTAAAGCATGACCGTCAGCATTGC
CCACATCGTCATGCCACTTGTTATTTTTGTGATAAATCCGGACATGTCGAACAAGTTTGTTCTCGGAAATCACGTCACAG
TTCCAGCTCGAAGCATTCAAAAATAGAACGTATGGAAATATGTGCTCTCAATTCTAACATGCGTGACTCAAATGTTGCTT
CTACCAAGGCTCATACAGACTCTAATTATGCTATAGTGTCACCTGATTCGCGAGTGCAACGCACTGCAAACAAACTTTTT
GTTACTTTGAACATTGCAAAGCACAATGTGAAATTTCAATTAGACACGGGTGCGTCAATTACATTAATAAACTCGGACAC
TTATCGTTTGCTCGGTAGCCCTCAGCTCTACCCGACAAACAGATGTGTAACTACATATGATGGTTCCGAACTTGATATTT
TAGGTGCGTGTTCTTTGTTAGCAACCTACAAAAACATTACAAAAATGGTTGAATTTACAGTGTTACGCGCGGCAAAACGC
CAAAACATTTTCGGCATGGACGCTTTTCAATTATTTGGATTTGAGATTCATGATTCAGTGTTGCGTGTGCAATCTTATGA
CATTGAACAAACAGTTTCTTCTTTGTGTACTGAATACGCAGATCTTTTTGATGGAACATTAGGCCGTGCTACTGATTATG
AGGCCCACATTACGCTTCGTCCCGATGCGCAACCTAAATTTTGCCGTGCACGCCCCGTGCCCTACGCATTACGCGAAAAA
GTCGCTGCTGAATTACAACGCCTTACAGACATTGGAGTGCTAGAACCTGTTACGTCTAGCCAATGGGCGTCTCCCATAGT
TATTGCTTCAAAGCCAGGTGGTGACATTAGAGTTTGTGTAGACTTTAAAAGAACAGTGAATTCTCAAACAGTGATTGATT
CCTTCCCGCTACCACGCGCCGAAGAACTCTTTGATAAACTTCAGGGTGGAAATTTTTTTTCTAAGATAGACTTAGCTGAA
GCTTATTTTCAAGTGCCTATTGACGCTGCTACGCAACAATTTATGGTGCTCAATTCACATGTTGGACTATTTCGTTTCAA
AAGACTACCCTTCGGTAGCTCTTCAGCACCAGCTTTATTTCAAAAATACTTACAGGGAGTGATTTCAAGTGTGCCATGCT
GTGCTACTTATTTAGATGACATTATAGTATCAGGACATGACTTTGATTCACACGTGGCCAACATCAAGAAACTTTTTTCT
GTTCTGCGTGCTGCTGGACTTAAGTGCAAAAAAGACAAGTGTGCATTTTTCACGCAGGAACTTGAATATCTGGGACATGT
TATTAACAGTCAGGGGATCCATCCCGCCCAGAAACATTTGCAAGCAATTAAAGACCTCCCAGTGCCTCGCAACCTTAAAG
AATTACAGTGTGTGTTAGGGAAACTGTCATATTATATTAAGTTTATCCCGCACGCAGCGCAAATTGCAGCGCCACTGCAC
AAATTGCGGCGCAAAAATGTACCTTTTGTTTGGTCTGATGCTTGCCAGCGTGCTTTTCAAAAGCTCAAAGACTCCCTGCT
CAGTGAACCTTGTTTAATTCAGTATGATCCCGCCAAACCAGTAGTTCTGGCTACTGATGCTTCTTCGCATGGCGTAGCGG
CAGTGCTTTCTCATGTTGTCGGCGCAACTGAGCGCCCTATTGCTTTCGCATCAAAAACGCTTAATTCTGCACAACGCAAT
TACAGTCAATTGGAAAAAGAAGCCTTATCCATCATTTTTGGAATTTCTAAGTTTCATCCTTATCTATACGGGCGAAAATT
TCTTCTGGTCACCGACCACAAGCCTTTAACGGCATTATTTCATCCCACCCACCCTATCCCTGATAGGACAGCGCAAAAAC
TTCAGCGCTGGGCTTTACTTTTGACGAATTATGATTACGATATTGTCTATCGTGCATCCTCCAAGCATGCTAATGCAGAT
TGTTTGTCCCGCCTACCTCTTGGTACGGATTTCGATTTTGATTCTTCGGAGGATTCGTGTCATCAGCTGGATCACGCTGA
TCTCGACACTTTGCAAACTTTTCCCGTTGACGCTCGTGTTCTCGCTTCTGCCACGGAACGAGACCCACAATTGAATCTTC
TTGCACGTTTCATTCGACGTGGATGGCCTGCTAATCGCAATTCAATTTCTGACCCACTTGTTCGTCGTTATTTTTCAATG
CGTCACGCATTATCTATCCAACGAGGTGTTATTCTAGTTCATTCCGACTCTGGTGCTAGTCGAGTTCTTGTTCCCAAACA
ATGTCAACACGCCGTTTTACAGATGCTTCATCAAGGTCATTGGGGTGTTGTTCGCACCAAGCAATTGGCACGACGCCACT
GCACTTGGTTTGGTCTCGATAAACAAATCGAATCTCTTACAGCGCATTGTCGCGCATGCTCCGAGAACGCAGCTTCTCCT
CGCCAGGAATTTTTCTCTTGGCCTAAGCCTTCAGGCCCCTGGGAACGTGTCCACATAGACTTTTGTGGCCCGTTCTGGAA
CACTCGTTGGCTCATCGTTGTAGATGCCTATAGTCGTTTTCCTTTTTGTGTTCCCATGCATTCAACGACTACAGCAAGCA
CCATCCGGGCCCTCGAATCTATTTTCTGTCTTGAGGGTTTACCGTCAGTTATTGTTTCCGACAACGGTCCTCAGCTGACT
TCTGCGGAATTTCGTAACTTTTGTTCTTCGAATGGTGTGCGTCACATTACGACCGCCCCATTCTCGCCTCAAAGCAACGG
CGAAGCCGAACGCTTTGTTCGAACTTTCAAAGCTCAAATGTCTAAATTGCGGAACGATCATCCCCGTGATCGGGCTCTCT
TGATTTTTCTTAGCTCATATCGTTCTTTGCCTTTCGGCGATGCTTCTCCCGCCGAACTTTTGCACGGTCGTGCTCACAGA
TCTGCGTTGTCTCTTCTTTCGCCACGCAACGTTCCGCCCTCGACATCGTCGTTTCATCCGAAGTTTTCGGTCGGCGACGC
GGTATACTTCCGCATCTACAATTCTTCGCAGCGTTGGGCTCCCGGCGTTGTCGCCGCTTCTGTTGGCCGCGCACATTATC
GCGTCTCTGACACGCGAGGCTTGCTGCATGCGCGCCACCAGAACACCTTGCGGCAGCGCCACATATCTGGCCTGCGTGAT
GCTGCCGCCGGTTCTATGCCCACAGATTCCGGGTCCATGATGACAGCACGCGCAGGTGGGCGCCCGCCCCGCAGCGCCAC
TTCTGGTCCCAGCAGGGCCGCCTTCGCGCCGCTGGCGCACACAGCTGGAGCGGCCCCGCAGCGCAGCCTGCGGTCCACCC
CACGGGTCGTCGCTCCGCCGTCGGACGCAGCCTCTCCAGTGGCCGCCGCGCCGCTGCCTCCGACGCTCCCGGCGCCATCC
TCGGTGCCACCGACGCTGCTGGCGCCGCCTTCGGTGCCCTCGCCGCTGCCGTCGCCGCCTCCGGAGCTGCCGTCGCAGCC
ACCGCCGCTGCAGTCTTCGCCTCCAGCCCGGCCGATGCCGATGGAGGTCTCCCCGGACGCCTCCCCTTCGTCCTCCTCTC
TGGCGCCTGGAGCCTGGGGACGTGCATCCGAGTCCGTTTTTCTACGTGGGTTTTCCGGCCTCCCGCGCGGCGAATGCCGG
GATGCGGACCGGTTGGCACCCACGTCAGCCACGCCACCGGCCCCAACGTCTCCGTCCCCAGGCAGCCCTCTCCCCCGCCG
CCGTCGTACAGCATCCAGACCGTACACGACGACGGCGCGGCAATTCACGGGGGAGGGGTGTAGTGAAGGTGCTGTCGATT
ACGTCGACAGCGCACAGAGGCGCCAGAGATATCGTCGCCGCAGACGTTAAGTTGGCAACAGCCGACTGATAACCGCAGGG
ACTCGCCAACCAATCAGAGGCCAGCGCGAGTTTTCCTGCTGCCGCGACGCTCCACATTAAACAGTTATATCCGAGTCCTG
AACGAGTTCTATTCGAGTACTATCGAGTCCTCAGCTTCGAGACGTCTTCCTGCACGCATCACAACATTAGGCCTCAGAAT
AACTTTGTCTGATATTCATACTCAGATAGAGTGACAGTTCTTGTGTCACTTAATTTATATTTCTCCGCACCTAATTCTAC
AAGATAAGTATTGTATATTTATATCAGAGTAAATAAAGTGTATATATATTTGTTCAGCTGTTCTAGCTCGCCGACACCTC
CGTCATCTCCTTTGCTCCCACAACAAAATAAAATTGTGTTGGTGCCGACAAAGCATCAACACAACACTGGCGCAGTCGGT
TTTTGGAACCATACTGGAGCAACAACAGCAAATTGAGCTACAACATTTGCAGTTCATCGGAGTGTCGCAGCAATACATTG
AAGCAGTCACGCTAACCTTGTATTTATATTTTTCAGATTGAGAGCACAGCGCAAGACTCGCATCAGAATAATTTTTTGTG
TGGCTAACAGTATTTCTGTTTCAGATCCAGTCCCTCATAATTTATATATATATTGTATTCTTAATTTGTTCCTTGTGCTT
AGTT

LTR-02

| Upstream Primers (5’-3’) | Downstream Primers (5’-3’) |
| --- | --- |
| ATAAACTGCGGGAACGAC | AGAAGACTGGCAAACTGA |

TGTTGATTTACATGTGTGTGTATTTTTTTTACACCTTCCTTGTGAAGTGTGGCTTTTGTGTCTCGTGTTTGTTTTCATTT

TTGATGTGTTGTGGTTTTATTATACTGCTGTTTTGTACTCTTTTATGTGAGATGTACGTTGAGTTTATTTTGAGTGGGGA

AGTTTTACTTTTATGCCAAACCATCCCGTGGTTTTGAGCTAAATTTATTTCGGATATCTGAGCATTATATACTCCACAGT

GTTACGGGAGATCTTTAATTGTCCCTTATTCTATCAGGCAAGGTTGACTGGTTTTACAAATGTGTCTCGTTTCATTCTGG

GGAAGCGTAAATTTAAGGGTTTAGGATGTTGGTGTTGCGACGGTTATGTTTGACAATACAGTACCGCTGTAATTGTTTTG

GCCCTAGTTCCGTATTGAGACGGCTAGAGGTTCTTATGGGCATGACATTGTGTACGCTGTCCCGCCACAGATTGCGTATT

AGGACATTGGTGTGCACATGCATGGTCCATTTTGGGTGTCGCGTGGGATTTTAAGTAGCCAACGTAATGCGTTTGGTGGG

CTTAATTAAAGTTGAAACAAGTTATTTGTTCTCTTTTGATGAGAGATACTGTTTTCCGCATCCCCATGAATGTTGCCTGA

TATCATTTGTTCTTTTCATTTGACCGAATCTGTCTGATAACTTGTCTGTGTCATGATAATACTGGAGTTATGTTTTTGTG

AGTGTCATAAAGTATGTCACTAATATAAGTTGTTTTCAGATATCACCGAAAGTTTTTCCTCACCCATTGAAGCAGAACGA

AACGGCCACGTTTTTTCACATCAAACAACGAGACGTTCTCCACACATGAACGGACGCATTATTACTGAGATGTGCAGCAG

ACGTCAGGACGACTCATTAATCTGAGATTTTTTTCCAGGAATTTTTTTTGTAAATGGTGGATTCTGGGACACCCCCAGAT

CTAATTTATGTATACTGATTGTGGTCCTGCATGGTTAAGGGCATACTCGAGTCTCTTGTTTTGGGTTAGCACGCTTGCAT

GCTGATTTAGATTTTAGTACTTGCTCAGTAAATGTACTTTGGCATGATTGTAACATGTTATTGATTAGTTTTATGGTGCA

TGGTAAGGTTTGAGTTATTTCTGTGTGTTGTTTGATGGCGCCAGTAGTTTTGATCTAACATACTAACTCATCCCCATCAT

ATGTCCTCGTGTCTTTTTAATATTTACGTGTTACCAAACACTTATGGATTTTTTAAATTCTGCAGAAATGGAAACACGCA

GACCGGAAACGCACTGAAGCCAGAATGCGAAAACGCACGGAACGAGAGCGCCAGAATCAAAATTGCTGGAAAACGCCTCT

TGAAGCTGCCTTTTGGGACGAGACAGTTTGAGATGGACGTGGCGCGGCCTTGTCTCCGGGCCCGGGCTGTCGCTACTATG

AGGAACCGTTAAGGCTTTGAAAGGGTAGCCTGGCCTCGCCAGCTGGCCGCCCGCCGTGGCTACCTGCCTCCACTGGCCTA

TTGTATTTTGACGGCACGCGACCTGTGTCTGTCAGCGTCCGTAGCGGTCCCACATCAATGGGGCGAACAACTCTGGAACG

TGTTTACAAGTTGTAAGCTGCTTGCCCTTTGACGGCGAGGACAATGCCTCCTGCACAGCGAGGCCCGTCTGTCCAATGTT

ACGTTCCGCTTCCCGACACTCGTCGATACCCCTGTGAGGTTAAGTGCGTCTAATGTTCTCATTGTTTGGCCAAGACATAT

GTGTGCGTTTGTTTCCGAGTCAGTACTGTACGTTTCGCGTGTGCAGAATAACTTTTGTGTGCTAGGACTGTGTTTTTGTC

AGCGGACATTCAATTCTTTTTCGTGTCATTATTTTCTTGTCATTTTTATATCATTGCATTGTTGATTTGTAACTGTTCAT

TCTGTACAGATACACGTGCCCTTGGCACATATGTAATGTACAATATTTATATACATTATGTTTCAGATTTAGTGTTTTTT

ATGTGGAACTGTTCCAGCTTTGATGTTGTGTATTTACAACTAGCAGATATGTTTTTGTTGCTATGTTTTGAGTGGTTCTA

ACCCTCATGAGTGAATGTTACTGTGTCGATTTTATGTGTTCTTTTTATTTATGTGTTCTTGTGTTATGTATACATAAAAT

TCAATATTTCCTCCCTTATGCCTTGGATGTGAATGGGTTGGATGAATGTGTTACTTGTCAGGGACATATTGTAACGAGAG

ATAGCCCATCAGTGTGTGATTGAGTTATAAAAGCTGTGTGGATCCACGGACTATAAACTCTCACAATACCACACAGAGAT

TGATTGCGAGATAAATTATTGATTTTTAGTAGAGAAATTTTTGTATAAGGTTTATCCTTTCTCTACATTTTGGTTGGCTC

GGCCAAGGCATTAGTCTTAAGTCAACACATTGTCATCTTGCATAGTGTTAAGGTCCCCTTGCATTTGATATTTGATTTTT

AATGATTTTATGTGTTTTACTTGTTCCCCCATTGTTTTGGTTATCTGCACATGGGTCCAAGTTCTGATTTCGTGTTCTAG

TTTGGGGAGTGATAGGGCTTCGTTTTTATTTTGATTTTCTTAAAAAGTGTTCAACAGAGTCCGACACATGAATATCTTCT

TGTGTCCAACTCTGCCTTCACACTTTTTCGCGCGGAGTTGGGGGTGATGAGACGTTTCTCGACATACCCATGATTTCGAG

AAACGCTCATGTTTGATTATCGGAGGTCTGCGTAGGCAGAATATGGGAAGCTTCCCACACAGAGAGAGAAACCTTGCTTT

CGAGTGTGACAGAGTGGACAGCTTAAGCTCCGAATTCTGTGGAATTCGGAGGATTCTAGGAACGCTGGCACTTGTGGTGA

GTTATGTTCTAAGGCAGTGATAAACTGCGGGAACGACAGTCGCCGTGTGAAGGACGGCCTAGCGGCAAGCCCCACTTGAG

AGGATTAGCTGGAGTGGGCGCGCAGGCACGTTTCCTAGCGGTTCCGTAGGGATTTTTCACACGGGGCAGCGTGTGACCTC

CACGGCCGCAACGCCGTTTCGCGTGAACCTTGGAGGGAAACGCTGCAATTCGGCAATCCAAAATACCGTCAGTTTGCCAG

TCTTCTGGTTTGGCGGGAAGACTTGTGATTGGATCTTAAGAAATAGCCCGTTTCGGGATTGGCCTGCGTCGGGTTTGTCC

CTGGCTCGGTACCTCGGTGGAGGGGTGTAATACGAGCAGGCGACAGAATCATACGCAGCCAGGAGAAGTCAGTCTGAGCT

GAGTGCTCGAGAGGAGAAGTTGGAGCCCAGCGCTCGATACGAGAAGGTCGGAGCTCAGCGCTCGGCAGGGTAAGTTGTAC

GGCGCAGTGCTTAGCGAGGAGGAGTCTTGGGACTTAGGTTTTGTGCGAGCTGTGTGCTCGTAGCTGATAGACTTTGGGTG

ACGAAGCCAGACTTAGGATCTGACTGATTTTGTGAGTGCAGACGCGGCCGCTTTTGAGCTTGCGCCATCTGTCTCTCTCT

CTGTGATTTCGTTTACGTCGCGACGTACCTTGTGGGGAGTTGCGGTCGCTTTCAGCGATACATAGGGAACCTTGTGCAGT

TAGCCTAATTCAGATTTTCCCCAGTATAGTCTGTTCTGTCTAAGTAAGACTTAGAACAATTATGTATACTGTGTTCCTGA

ATAAATGCTAATATTTACCAGAATTTTCTCCACATCATTTAGTAGGATAGAATTCATATCACTACTAGATAAGAGATTTA

GTGATTTCGCCAGCCTCCCTCACTGCTTGTGTGTACCTGTTAAATCGTGGTAGATAGCTTTTCCGTTGCGCACAAGGCCC

GTAATTACTAACAGTTTCCCTCCGTGGTATGTTAGGCAACAGATACGCACAGCAGGAGTCTCTGCATTGACCCTCAGTCA

AAGCACTACTAGAGAAAAGAGGTTAGGTGTTATAAATGGCGTACCGCTGCCAGGATTTTCGTTCTGAAAATCTTTATTAA

TGTTGAGTTCCTTGAATTATTCGGGTTCCATGATTAATTATTAGCTGCAAATTTAATGTCCCTCCCTTATGCTGGAGTTA

GGAATAGCGGCGCTATTTGCTGATTTATGTGTTTTTTCTTTTCAGCTGTCGTGGAAAAGCATTTCTGTATGCCTTTTTCC

AGTTGTTTTTGTACATCTGCGTTGTAAAACAGCGTTGAATTTTTGTGTTTGTGATTTCTCTGTCTTTGTGTATATGTGTC

TTGATATATGTGTTTTTATATGTGTGTTGATATATGGGTGCTGTACCGTGTTTGTGTTCGGTTTTTGTGTTAATTTGTGT

CGGCAATTTTGTGATTAATTTGTGGCTAGGGGCCGCGATTTTATTTATGGATGGAAACGCGTAGTAGGACGCGTGATAGA

CTGAGACACGCCAGTCAATCTGATTTAGAGGCAGTTTCCGGGGGCGCAGAAGTGCTTACGGAAAAGGGTATTGAAATTAT

TAATACGAGGACGGGAATAGTTAACGTCATGGACGCCGTCGGTTTAAACTCTGTTAATGAGGAAGCTTTAAGGGAACCAG

AGCAGGTCGATCCCTCAGCTCTTACACCTGTCGCGCCAGAATGTAGTGGAAATGTTGTTCCACGAACACAGTCGGGAGAT

TCCAGTGGTTTAGGAAGCGGAGATTCGGTCGCAGCGACCGTAATCACGGAACAACCGGCGCAGAACTTGAGTGTGCCGTC

CGCCGCTAGTGTTACGAATGACCCATTCGCCGCACTGGCTAGCATGATTAAGGGAATGGAAGAGCGAATGGAAGAGCGAA

TGAAACAACTTGACCAGGGCCAGCAGGAAATCGCGTTGCGCCTGGGAAAATTGGACGAGTTTGGGGCGAAAATAGACGAG

CTCCGCTCGGACCATCAAACCCAGTCTGCGAAGTGCACTGCACTTGCTGAAGAGCATCAGCAGAAATTTGCCGTGGTGGA

TGATAAATTAGAGGGACTAGCCGCAGAGGTTAGCAAGGTCCACGATGTTGAAAAGGATGTGTCTGCTCTCGTAGCGCAGC

AAAGCGTCCAAGAGAGGCGTGTGGAAAGGGCGGAATCTCGCGTCAGTGCGCTAGAAGATCGCCTCGCGGATTTTCCGGAG

AATATCGCGACTAAACTCGCAGAGGCAAGAAACGAACTTTGCGAGGAAAACCGGCGAATTCTGCGAGACGAGGTTAGGGC

CGCACGGCTAGCCTCAGAAGGTAGAGACTGTGAGCACGCGACAGTTAACGCCGGACGCGCAAATGTGGACACCGCTTTTC

CGCTGCGCCGCTGCGGACAAATCGATCGTGAGGGGACTGAAACCTTGTCTCCGACGGTTTCTGGCAACAACAGCCAAAGC

GCTGTGAGTGGGGAGCACGATAGATTTGAGCGAACGGATGAGTTGTTCGATTATAAACACTTCTTGTCCATCCGCAAATT

TCAAGTGTTCCGTGACAACGCCAACGGTCCACACCCGAAGGCGTGGGTCGAACAATTCGACCATAATCCTCCGTCCACGT

GGCCAGCCGCGCGAAAAATAGAGTTTGTCTGCGGTTTCCTAGAAGGAGACCCAGCGACAATTATGAGAGCCGCTGCGAGT

GACTGCCATACATATGAACAGTTTAGGGAAACCTTCCTAAGGAACTACTGGTCAAAGGCGGCACAAAATCGAGTGAAACA

GGCGATCATCATGGGTCATCCGTTCAATGACGACGGGAAAGACGGACCCGTGAAATTTTTTGAGTCTATGTTGCGAAAAA

ACCAGTTCCTGGACAATCCGATTGCTCCCAGTGAACTTATTGGTTTTTGTTTTACAAAGCTCAGCTGGAAATATCGCCAG

CTGCTCATTGGCAAGTGCGGAGACGATGTTGAATCGTTTAGAGCTACTCTGCAAGAGCTAGAATTTGCTCGAGACATAGG

CGAGCCGCAATTCTCTGGGCCGCAAATGCAGCAACAGGCGCAAGGCGCTTACCAGAACAATCGGGGACAAGGTTATACAA

ATAACCAGCAAAATAGCTCGAATAACCACTTTAACAAAAAAGGGAAGTGGAATAATCATGGAAATAATCAAGGCTTCCAC

AACAACGGTGGAAATCAATGGCATCAGAATGCTCCGAATTTCCAAAATAGCGGAGGAAACCAGTGGCATACGCCTCCACC

ACAATTCCACAGTGGGGTGGAAATTAGGCCACCTAATCCGGTGCTCAATGGGCAGGCTGGTACATCCAGTCCTGAACAAT

AAATTGATCTGGCAATGCAGTTATAATCTGGTTCAATCTTGTGGTTGAATGCGGATACTCTGTACAGACTTAGTTTAGAT

TAGATTTTATGAGATTTTGATATATGTGTGTTTTATTTTTGTACCATGCTGAAATTTTGCATGGACTGTGTTTTAATTGT

GCAAGCAATGCACTGGAAGTCGAGACTCTGAGGTCGCGCAAATCACGCGGACTCCATCACATTAGCAATGTGAAGTTGTT

CTTGGAGAGATAGCCGTCTGATGATCCATGTTGTCCTACTAAACAACAGCTGTTACATTTTTGTCTGCTGAGTTTATTTT

TTTGCGTCTTTTGTATTTAC

LTR-03

| Upstream Primers (5’-3’) | Downstream Primers (5’-3’) |
| --- | --- |
| GTTTGCGGTAATAGTTCACAGTTCCT | TACTTGTCTTTAGCCCATCACTTGC |

TGGCGTGCAAGCCACCAAAGACAATACATATATCAAAGATATTCGTTGGTCGAACGTCGATATTGTCAATGACTGGCGTG
CCAGCCGCCACACAGCCCCCGCCCGCAGTATGGAGTACTACTGCGAAGGAGAGAGAGGACTGGCAACCAGCGAAGATGGT
GGCGGTGATGTGATGGTGACTGGGATGGCGTGTGCACCACGAGGCGGGGGCAGTGCGCCGTCGACAAGGCGCAACGCCGG
TCGTGGGTGCACGAAGTCGTCGAGGGCCGCTGGTCGACGTAGGGGGCGGCCGAGGCGAGATGCTGCACTGTAGTACACAC
GCAAATACAGCCTACCATCGTCGCACACGAATGCTTGCGTTTTTTCCACCAGGCAAGATGGACGGAGGACGAAGGACGTC
ACACGAGTGGGGCGAGGCGACAGCGAATCGTCGGTGGATTTCGTGACCAGGACGTTGGCCAGTCTATCGCGAACCACCAC
GGAGATGTACGTGATATCGTCGGGCGGAACTCGTGTCAGCAGGTCCTCCGATGTTATGACGGCTCTGTCGGTCGTTGTGA
CAGCGGGGGCCGTGTCGAAACCGAGGAAGGGCGATGCTGACGAAGAGTCGTCGAGAGAACCCGAGCAGCGGGCGTCGAAG
CTGGGACTGCTCGTGGGCGAAGTCGAAGGCGAAGCCGAGACGTTGTCATGAGGACCGATCGAGGCAGGACAATCTCGAGG
CGAGCCGGAAGAGGCAGAGGTGGCCGAAGAACCCAGGTCATGGGGAGAGGCCAATGTGCGCTCGAGGCCGGACGAGGCCG
AAGAAGATAGGTCTGCTGAGTCAGGAGAAGACAGGGCAGACGGGTCGACTGCTGAGTCGACGAATGCGGGTTTGACACGA
TGCAAGGATACAGTGGTGCGTGATCCTGCGATGTCAAGATCGATGGTTTGGTGCGATCGGGAGAGAACACGATGAGGACC
CGTGTAGGGCGGATGAAGCGGGGGCTTGACCGTGTCGTCCCGAAGCATGACAAAGTCACAATCGCTGAGAGCCCTCGGAA
CAAAAGGCGTCGGAGGCGAGTGGCTGCGAGGCGGTGGGATGCGCAGCGCCTTGCAGTGGTTTCGAAAGAAGTCGACGTAA
GTGGGCAGATCGACGTTGTCAGGAAGAGGAGAAGGCTGAACAAGTTCCCCAGGTAGAGTTAGGGTTTGTCCATAGACAAA
TTCAGCAATTGTCCCCTGCAAATCCTCCTTGAAAGTGGCCCTGATGCCGAGAAGCACGAATGGGAGCGCTTGAGACCACA
TTGTGTCGTGGCAGCGGAGGGCGGTTTTGAGAGTGCGATGCCACCGTTCTACCAAGCCGTTTGACTGAGGGTGGTAGGCG
GTGGTGTGCACGTGGGTGATGCCGCAGATTGCGCAAAGCGATTGGAAAAGAGCAGATTCGAATTGTCGGCCTTGGTCGGA
CGTGACCGCGGCGGGACAACCGAAACGCGAGATCCAGCACTCGAAAAGAGCTCGGGCAACAGTGTCGGCAGTGATGTTAG
TAAGGGGAACCGCTTCCACCCAGCGGGTGGTGCGGTCGATGACAGATAAGATGTATCGGAATCCCTCGGATATCGGAAGG
GGACCGACGAGGTCGATATGAATGTGTTGAAACCTCGCAGATGGAGGTCGGAACTGACCAAGCGGAGGAGTGGTGTGACG
ACCGATTTTGTTGCGTTGGCAAGTGATGCAGGAGCGGGTCCACTCTTGGCAGTGTCTTTTCATGTCCCTCCAGACGAAAC
GTTCCGAAACCAAGCGAGTCGATGCTTTGATTCCGGGATGAGAAAGGCTGTGTAGCTTTTCGAAGACATCGCGCTGGAGG
GAATGAGGAATGTAGGGGCGGAGACGGCCCGTAGACTCGTCGCACCAGACTTGGTCGACAATACCAGGAAAAGAGGTTTT
TACTGGCTTGAGAGAAGACGAGGTGTCGTTAATTAAGTTCATCAGTTCGTCGTCGGCCGCCTGTAAGCGGGCGAGGTCTG
ACAGGTCGATTAATGAAGTGATTGCGCCGACGCGTGACAAAAAATCGGCGACAACGTTGTCCGCCCCCCGAATGTAGCGA
ACGTCAGTTGTGAACTGAGAGATATAATCAAAATATCGAAAACGACGGGGCGGAGGATCCTTTGGCGGGTGTTGTATTGC
AGAAGCGAGGGGCTTGTGATCGGTGAGAACATAGAAATCACGACCGTCGAGCTCTGCTTTGAAATGTTTTATAGCCTCGT
ACACGGCTAACAGCTCCCGGTCGAAGGCCGAATACTTCTGTTGAGCGTTGTTGAGCTTTTTCGAGAAGAAGGCCAAGGGG
GAGGTTGTGCCGTTGAGAGTCTGGCTGAGAACAGCTCCTACTGCCACGTCGCTAGCGTCTGTGGTCAGAAAAACTTGTGC
ATCCGGGTGAGGAAACGCAATAGTGACTGCGTTGGCGAGCAGGCGTTTAAGGTTGTCGAATGCGTGTTGCATGTCCTGCG
ACCAAGGTACCTCACGGGTGCCGGAAGTTTGAGGGCCAGCTAGTGCGTCGGTTAGCGGTGCTTGGATTTCGGCAGCAGCA
GGAAGGTGTTTGCGGTAATAGTTCACAGTTCCTAGGAAACGTCGAAGGTCTTTGTACGTTCTAGGACGATCCATAGTCAG
TATGGGTTCGACTTGAGCCGGCGGAGGCGAGATGCCGTCCGCGGAGACGTGAAAACCGAGGAAAGTTACAGAGTCTTGAT
AAAGCTGAAGTTTTTTATCGTTGACGTCGACGCCGGCTTTGCGAAGCGTGTCAAGGACGATTTGGACGTGTCTCTTGTTC
TCGGCAAGTGATGGGCTAAAGACAAGTATGTCGTCGAGATACGTGAAGCAAAAGTCTAGGCGGAACATGACTTCGTTGAT
GAACCGTTGCCAGGTTTGTGTGGCGTTGCGAAGTCCAAATGGCATGCGCTTGTAGTGAAAGAGGCCAAACGGAGTGGTAA
TGGCTGTTTTTTCTACGTCTTCGGGTGCCATGGGAATTTGATGATAGGCGCGTTTACAGTCTATCACGGAAAAGCAACGA
GAACCAGCAAGAGAGTTCGTGAAGTCGGCGATGTTCGGTACCGGGTACCTGTCGGGGATGGTGCGGGCGTTGAGAAGGCG
GTAGTCGCCGCACATCCGAAGAGAACCGTCTTTCTTTTTGACAAAGTGAGCCGGAGTAGCCCACGCACTGGATGATTTTT
CTATTATCCCGTTGTCCAGATGGATCTGTATCTGTTCTTTGGCAAGAGGGACAAGTTCTGGTTTAATTCGACGTGGACGA
CAAGCGATCGGAGGTCCGTCGATGAGATTAAGTTTATGTACAGTGCCATTCGTTACGACCGAAACTCGTGTCGTGGGCGT
ACGGATTGAAGATTGCTGCACGAGAGAGGTTGTCACCTTAGTTGAGGCATGTGACGCCGGACGAGAGACAACCGAAGCTG
AAGCGTGAGCGCGCGCAAGTGGGCGCGGCTGTGATGGTTCGGTAGCAGCTGACCGGGCTGCGAGAGGTGGGCGTGGCACA
GTGACAGCTGCGCGCGGCGTCGTTCTACTGTTCGTGGTGGCAGGCGCGTGAGTTGCGGGCGCTGCGGTCGCGCAGGCTGT
GGAGCCGGCAGGCGCTGCCAACCTAGGCGGACGTGAAACGCTACTGTTGCCGTGCTGAAAGCTAGGTGAGCGAGAGTTAC
TAGCGGTCTGTTTGTTATTGTTTTTGAACTGCGAAGGCTTGCCGTGCACGGACGTACAGTTGCGAGCCGGAATTAAGAAA
ATGTCTGGGCGCTCGCGAAAGGTAACACGTTTGGTGACAATGTTTGTAGCGCCGGACGAGATTGCTTTGCTGTTTGCAGT
GGGCGTTGCCTGGCAAGAGCGAGTGTTTTGACAGTTCAAAAGTGGCGTTGGAGCGAGCCTGACAGTAGGCGGAACCAGCG
GAGCCATGAATTTAGAGTTCAGAGGCGAGGTTACAGGTTGAGAAACCGCGTGAGAACTAGTTGTCGAATTGACATTGTCT
GTTCGTTTACGAGCGTTCGGGGCCGAAGAAAGTTTGACAGCCTCCGCGTAAGAAAAAGACGGCACACCAGAGTCACACGA
GTGCGAGTCAGCACGAGACGAAGTGGTTGCAGGGGGCAGTTCACACTTGAGCACACTGTGACTAACCTGGTTGTCTTTGA
GATTGTGAGCAGCCCGCAGCGAGTCGAGGCGACGTTGCAGGTCGGTGAGCTCGGACGACTTGGTCAGCAGGCGCGACTGC
AGGTTGACGTTCTCGCGCATGAGACTGTTGCGCTGCTCGGAGACGGTGACGAGTCTCGATGACGCCTCATGCAATGACAG
CGACAGCTTTGCATGAGTGGCGATGAGGTCGGAGAGCTCGTTCGGTTTGGTTGAGAGCTCGGAGATTGCAGCGCACTGAA
AGGTAGTGTTCGTAGTGCTGTGATAGATAGAGTTCGAACCAGCGTCGATGGCAAGCGCGTGGTGTTTCAGGAAGTCGATG
CCGAGAATAGGTTCAGGCACGTTAGCGACGAGAAAGGTCCACTGTAACTTGCAGTCTGGAGACACATCAATCGAAAAGTT
CGCCGAACCAAGACACTGGAGAGGCGTGTTGTTTATAGCTTCGAGTCGTGTTGCGTGCTTTCGAGTCACGAGGTGTTTGA
AACGAAGCGGCAACAGAGACACATCAGCACCCGTGTCGACCAGGAAGTTGAGTCCGGTGACGAAGTCTCGTAGGTACATG
CGGGCACGGCCAGTGCTGTCAGTTGAGACGGAGTGCAGGAGGGCGTGGCGCCTGTCATCGGAGGAGCCGGAGGTGGCGCC
TGTGCCTACCGGCTCGCCGAGTTTGGGTGCTCGCAGGGCTTGCGGCACTTGATTGCGTCGTTCCCATAAACTCGATGATA
GTAGCAGTATTTATGAGAAGTGCCCGAAGCTGCGGCCTGAGGTCGAACCAGAGGAGGCGGACCTGACGAGCAACTCGAAG
GTGAGTCTGGTCGTTGTTGTTGCGTCGTAGCAGCACGTTGTCTGACAGGCGAAAGTTCATGTGACGCGGACTGCTGTCGT
TGCGGAGGCTGGGTGCAAACTCGTTCCCAATGTGAGCTCTGCGTGTTATCCTGGATCGGCATCGTGTCGAGGTGGAGCGA
AAAGTAGGCTCGATCAGCGTGACAGATGCGATCGTCAGGAGGTAGAGCGATTTGAGCCGTTGCTGCCAGCTGAACAGGTA
GAGGCAGTTTGTCAAGCCACATGTGAAATAACGTCTTGTCAGGCATGTCGGATTCGCTTGCCATATTTCTCATCTTGACC
CAGAGCGTTGATGGCTTGTCGTCACCAAGGGATATGTTTTCGAGTCGTCGTAGAAGACCCTCGTTGCCAAATCGCGAAGT
TCGGCGTAGAATGTAGGATTTGGCAGCACGGTACTTGTCGTGTGGAGGCGGCGCACGAAGTAGTCCGTAAATGTAGTCTG
GGTGTTTGGAGAAGTGAGAGATGAGCGCGATGTATCTGGCGTCATCCGACAGTTTGTGGAAGTCCAGTATTTGTTCCACG
AGAGCGAACCATAACTCCGGAATCTCCTCGTTGAATTCAGGCAGAGTCGGAAGTTTGTTGGTGCTCGAATCAGCAGGTCC
GATGCAGGCGGAGCCTGATTCGATGAACGCGGCAGGCGCTCGCGCGGGAGCGGTTGTTGATCTGAGCGGAGCGCGAGTCG
TGTTTACATTCCCCAGTGGGGCGTGGCTGCCCACGTGACCGGTGCCTCGCGTAGGAATACAATGCGCGCCGGACGGTTGC
GCATGTTGCTGTGCGGCGGAAGCATCGTGGCGCACAAAGAAAATAGTCCCCGGAATTTTTTCGAAATAGTGCATTTCACT
CGGTGAGGAGGAGTTCGCAGACACAAAAAGTTCACTATTAATGTTCGTTGCACTCGACGGAGTGATAAATGCAGTCTGTT
GTGGGGGTGCGAAATTTCGAGGCACGTGTGCCGGTGGCGGCAGTTCGACACCTGTTGCATTCGAAGTATTTCGATGAAAA
TGTTTAAAGTCACTAAATGCAGTAGGTGGAGGTAAGTAAACACGAGAAAAAGAGTCTGATGTGCTCGGAAAGGTCGAGTA
GACACTAGCGGAGCTGGATTGTTCATTGTTCGGCACGTGGATGCACGACTGTTGCGTAGTTGTAGCACAGGTAAAAACAT
CGTCACGATTAAGAAAACACTGGTTTCTCGTAGTGTTATCGCAATGAGAGTTCGACGGTAGCGATGAATTTGATGAGAGC
ACAGTAGATGTAGCTCGATCCGTTCGGTGCGTATGTGGCGTGCTATAGCCGGAGTCGTACAGGCGAGAGTTCACACATGA
TGCACCAGAACCAAATAAATCACTAGAAGTATTGTAGGAGTGAAACGGCGTGCTCTGAGGTCGAATTTGATGTGAATCCG
TTGGAATGGAGACGTTGTTCTGGCCGGTGGACGCCGATCCGGTGTTGGCGGGAACGTTATCCATTGTCCAGGGTAGCACT
CGTATTTGAGACCCGAAACGCGACGCGGTTGACGCGACTTTCACTTTACGATACGGAATATTCGTTCAAAACGGAAGGGT
CACCAATTGAAGCGAGACTGAGGTATTGGGGTACCAGGGAAACACTTCTAGTTAGAACATACATTTATTGCATACGAACA
GTACAGAAACATAGAACCAACTGGCAATA

LTR-04

| Upstream Primers (5’-3’) | Downstream Primers (5’-3’) |
| --- | --- |
| GAGCTGGATATGGGCGGAGAAGG | TTGGCACATTGAGGATGCTGACG |

GGAAAGTAGTGAGGGCATCGTCCAAGGAGAGACCACGGGACAGTTTAGACATTTGGGTCTTAAACGTGCGAACAAACCTC
TCAGCCAATCCATTGGAGGCGGGATGGAAAGGGGCGGTATGGACTAGGTCGATACCCCGGGTAGAGCAGAAAGATTGAAA
GTCGGCGGAAGTGAATTGTGGCCCATTGTCAGTGACCATAGACAGCGGGAGCCCTTCAATGGCAAAGATGCGGGAGAGGG
CCTGGATGGTGTGTTGGGAAGTGGTGGAGGTCATGCGGGAAACATAGGGAAACCCACTGCCGGCGTCGACAACCACAAGC
CAGGAGGCCCCCAGGAAAGGGCCCGCGAAGTCCAGGTGGAGGCGAGACCACGGCGTCGTCACCTCAGGCCACGGGAAGGA
GCGGCGCGGCGGCGCCGATTTATTGCTCTGGCAGATGGAGCATGAGGCGACCAGGGCAGTGATGGCAGCATCCATGCCCC
GCCAGTAGACGTGCCGCCGGGCCAGCCGCTTCGTGAGCACGACGCCCCAGTGCCCCGTATGGAGGAGGGCAAGGACGCGT
TCCCGCAGCGACTGCGGGATGACGACCCGGACCTGATCGTTGTCCCCCTGGAGTAAGAGGACGCCCCGGAGGGACGAGAG
GGCGTCGCGGCGCGTCCAGTATGCCTGAACTTCTGGTCGGGAGATCTGGCGTCGGTCCGATGGCCACCCCTGCTGCACAT
GCCGAAGAACGACCTGAAGATCTGGATCCTCAGCAGTGCAGCGGGCGACCAGGGTAGCATCCAACGGAAGCGAGGCCAGC
GCCGCCGCTGCAGCGTCGTCGAGGTGAAAGCAGACGATGGGGTCGGCGTCGAAGACAGGGTCGGGTCCCACAGGCAGCCT
GGAAAGTAGATCAGCATTGGCATGACGGGCAGTACTGCGGAACTGGATGTCATAAGTGTAGGCAGACAAAAACAGTGCCC
AGCGCTGGAGGCGGCGGGCCGTCCGAGTGGGGACCGCCGCTGATGAACCAAAAAGAGAGACAAGAGGTTTATGGTCGGTA
AACAACGTAAACCGCCGACCATAGAGGAAGTCATGGAACTTTTTGATTCCAAAGATCAGAGCCAGGGCCTCTTTCTCTAT
TTGGCTGTAATTGCGCTGTGCGGGAGAGAGGGTTTTAGAGACAAATGCAATCGGGCGCTCCACACCATCCACCAGGTGGG
AAAGAACAGCCCCGACCCCATAGTCAGAAGCATCCGCCGCCAAGGTGAGAGGCTTGGAGGGATCGTATGCCATAAGGCAA
GGAGGGCGAAGGAGGCTCTGCTTGAGGCGCTGAAAGGAGGACTCACAGGCCGGGGACCATTCCCAGCGGACGTTCTTGCG
AAGCAAGCGGTGCAGGGGTTCAGCCAGCGCCGCCGCTTGAGGAATGAAACGCCGGTAGTAATTCAGTTGGCCCAACACCG
ACTTAAGTTGGGTAGAATCCGTCGGAGCAGGCAGTCGTTGTATGGCCTCGACATACTGAGGCATGGGCCGGATGCCCTCA
GCGCTCAGGATATGTCCCAGGTACTGGATGTGGGGAACAAAAAAAAGGCACTTGTCCTTGTTGCAACGGAGGTTCTCCCG
GCGCAGAACAGCGAACAGAGCACGGAGATTGCGTGCAAGATCTGGGGCATCCGTGCCGGTGACCAGAATGTCGTCCAAGT
AATTTGCGCAGCCGGGGACATCTCGCGTAACCGACTCGAGATACCGCTGAAAGATGGCCGGCGCGCTGGCGACGCCGAAC
GGGAGACGGTTATAACGAAAAAGCCCAAAGGGCGTGTTGATGACAAGGAGTTCTCGGGACGTGTCATCAAGAGGAAGTTG
GAGATAGGCGTCCCGGAGGTCGATTCTGGCGAAAAGGCGGCCGCCAGCCAGGCGAGAAAGTATGTCGTCCACTTTGGGAA
TGGGGTAGGTGTCGATCACCGACTGGGCATTTATGGTCGCCTTGAAATCTCCGCAAAGACGCAAGGACCCGTTGGGTTTT
TCGACCACCACAATGGGTGTTGCCCAGCGGCTGTGAGGAACAGGGGAAATGACCCCCTCATCCTCAAGGCGCTGCAGTTC
GAGGCGCAACTTGTCCCGCAAGGCGTAAGGGACAGAACGAGCCTTGCAAAATTTGGGGACTGCCGAAGGGAGGAGCTGGA
TATGGGCGGAGAAGGTAGAAACGCCCGGAGAGGAGGCCGAAAAAATATCGGAAAAATCAGTGAGCAAGGTCTCCAGGTAA
GCAGAGGTGGACAAGGAATGTACTGCAGTAGCAGAATCCTGAAGCCCAATCCCCAGTGCTCGGAGGAGGTCTCGACCCAA
GAGATTGGTGGCCGCCGGAACATTCACGACCAGGATGCGGGCAGAGACTGCCGTGGAGCGATAGCGGACCTGAGCAGAGA
ACTGGCCCAGGATATCAATGTGGTGGTTAGTATAGCTGCGCATGGGCGTCACAAAAGGTCGAAGGGGAGGGGCCCCTAAC
CGCCTATGGGTGTCCACATTGATAATCGTGGCGGAGGAGCCGGTGTCGACCTGAAAGGAGACCGCCGAGCCATCAAGAAG
CAGGGAGAGATTCCAGACATCGTCGGTGGAGGGAAGCACGGACCGCACAAAGGAGACCTCCTGAGCCGGCCCGTCAGCAT
CCTCAATGTGCCAAGCTGGGGCCTCCGTGGTGGTGCAGTCCACCGCCATGGCAGAGGAACGACAGACCTCCGCACGGTGG
CCCCGCTTGCCGCAAGCACTGCACGTGACCCAGCGGAAGGGGCAGGCAGCCCGCAAGTGGTCAGCAAAACAGCGCCCACA
GGACGGGAGGGCCTGGGGGGACCCCCGCTGTGGCGCCGCCGACAGCTGTTGCTGCTGCAGCTGGTGCTGCTGCGCCGGCC
GAGGGGACTGGTGCTGCGGCTGCTGTTGCTGCCTCCGCGGCTGGCGGCGACGGGAGGGGGCCGAGGCACCCGGAGCTGCC
GGCAGGGGCGGGCCGGAACCGGCTGGCCGTGTGGGGGGCGGACCCCACTCTGGCGGACGGGTGTAGTACACGCCAGAGGG
ATCGCGAACAGCCGCAGAGGTATGAAGCGACTGTTCAAACGCCCGCACCACCTCCAGACAGGTCTCCAGGGGAGGGTCCT
TCAACCTGAGGAGGTCCATCCGCAGGGCATCGTCCGGGGCATGCACCAAGATCATATCCCGGACCAAAGAGGAGCCGTAA
GAACGCTTACAAGCCGGGTTGGCACAGTGAAAGCGACAATCGCGGGAATAACCCTGGAGCTGAGCGACCCACTCCCTATA
CGACTGGTGAGGCAGCTTGTGACAACTGAAAAATTTGTGTCGAGCCGCCGAGACATGAAACTGGGAGTCAAAATAGTCAA
AAAGACTGGATTTAATGACTTCATACTGGAGGGTATGGGGCTCCACGTCGGGATGCAACTGCTGGAGCAGGTGGAAAATG
TCGGGGCCGGCATATGCCAGAAAGAAAGCACGTTTCTGGGAGTCATCGCTGATGTTATGAGCCACGAAGTACTGCTCGAG
GCGGGCAACGTAGTTGGCCCAGGGTTCGATCTTCCTGTCAAACTGTTGAAAAGCCGGGGGCGCGGAACGCATCCCGTGAA
CGGCAGCAGAAAGATGGGACAGCTGAGCTGAAAGCACCGGGTCGGCCGCAGAACCGGACGAAAGTCTGTTGCAGAGCATT
TCGTTGAATTGCAGCATCTGTTGTAAGATGGCCAAGACCGCGGCCGAAGGTTCATCGACGTTTTCCATCGTGGGAAGAGG
AAAGAACGCGTGAAGACTCAAAACACCTCGTCGCCAAATGTAGTGTCGGCGTCAGCCGAACAAAAGAATAAATACTCAAA
AGAAAGGTTTATTGACCACTTAGGCGGCGCGAGCCGCAAGATACAAATGAGAATTTACAAAAAAAGCACGCACTTCCGCG
TGATAAATAAAAGGAAACAAAACACAGTTCTCTGAACAAAACAAATGATTCTAAGGTACAAAGAGGATAATACGAAAATA
ATAAACGGCCTAACAGTCGTGCTGCCTAGTGCAGCCCTAACGGGCTCCGCTGCTGTAGGTACGAATGATGGGGAGAGTAG
CTCCCGGCCGCAGCCAGTACCAAGCAGCGGAGACCGCTGTCTGCCTGTAGACGGCGGCGGCTCTGCTTATATGGACCGCA
GGCGTAGGAATATTCTGCGTTTCCTCCGTGACACGTGGTCTGGCGGAAGAATGTAGTCCGCGCCGACAGCGCCATCTCGC
GGGAGTTGCTCGTCGGTTGGACTACGTCGAATGGAGAGTGCCTGTCGTCTTCCGTGTCCGCTACCCGCGTAGCTTCGGCG
TATGATGGGTAGACGACTTGGCCGGCGTTACTGCATCCCTTCCCCCCACCCCTCCCGCGCGAGAAGCGGAGAGGCAGAGG
CAACAAGCCGCAGCGGAGTCGGAACACCCGGCCGGGTGTTGTGGAGGATCGACTTCCATGGGCGTCGGGCCGTCGGCGGC
GGCTGGCGCAGGCGGGATGTCCCCTGGAGCAGGGAGCGGCACCACCGGAGCTGGGGGCAACGGCGCATCCCCCGACGGGG
ACGGCGGTTGCGGCGCTGGAGACGCTCGTGGCTGCTGGGCGAGGCGGCGCCGGATTTGGTCGACATGGCGGCGGCACAGC
GTACCGTCCTCCAACCGGATGACGGCCATCGACCGGCCGAGAGGAGCTTGCACTGTGGCTGGCACCCATCGTCGCCCAGA
CCGGGGGAACACGAGGGTCCACACAGGGTCCTGTGGCGAGAAGCGGTGACGGGCCGGCCGGGAAGGGGCAGGAGGGGAGG
GAAGAGGACGGAGCACTGAGAGTGGGGACCGGAAGGGGCGGCCATGGAGGCGCTCGGCCGGGGAAGAACCCTCCTGAGGA
GTCGACCGGTAGGTGGCGA

LTR-05

| Upstream Primers (5’-3’) | Downstream Primers (5’-3’) |
| --- | --- |
| CCACCTACTGGCAATCAAGAATC | CCAAGAAGAACTTACGACCATACAA |

GATTTCTATATTTGTATTGTATCTCATTGCAATTTACGTGTGTGTACGACATGGCTACTCAGGAGCCACATACTTTGCCA
GGTATTGATCTGCAAACCTTTTTGCAGATCCAACAACAAGAAAATGAGTTGCGTCAGCAACAAATTACGGCGTTGCAGAC
CATGCTGATGGAGCTTTTGCAACGACAACAACAGCCGCAGACGCCACTAGCTGCACCACACGTGACGTCACCTCCTGCCA
AGCACGCTGTACCACCGTTCCGTCCCTTTGATGAGCGGGCCGAAGAATGGGACATCTATTACGAGCAGCTGATTCACCAT
TTTTCAGCCCATCATGTCAAAGGTACAGCCAAGCTTACGCATTTTCTATCTTGTTGCGGCGCCTCGACTTTTGCGGTAGT
TAAGAAGCTGTACCCCGCGACGAACTTAGATACTCTTTCGTTTGAAGCAGTAGGCACGGCCTTAACAAACTATTATTCAG
AGAAAGTTCATGTCGCGGCAGCTCGTTTTGCTTTTGCTAGAACAAGGAAAAAGCCAGATCAAACTTATCGCCAGTGGGTT
ACGGAATTGCAGGGCCTAACCCGCAAGTGCCAGTTTAATTGTGAACCTTGTGGTGCATCGTACTCTGACACAATGATTCG
TGATGCAATAACTCACAATATTGATGACAGGAAAATTAGAGACCAAATTCTTCAATTTTCTAATCCTACATTAGAACAAG
TGTTAGCTATCATAGACCACAAAGATGCGTGTGACTCAGCTCAAGCTACTTTTGCTAATACTAACATTTGTAACGTAGAC
ACTGCTACTGTTGCTAAAGTTGAAAATTCTCGTGTGCGCCGAGCAGGCTCGAACTATAAACATGTCAACAAGCGCGGCCC
TCGCGCGCAGCACGCAAGTCAGCAGTCAAAACAAGCTCGCCGCTCATGTCCTAAGTGTAATGTTAAACATTCTCGTGACA
ATTGCCCTTCACGTAAAGCACAATGTTTTTCGTGTAACAAAACTGGACACGTGCAAAGTGTGTGTATGCAACGTAATCGT
TCTAACATTCAGTCGACGAGTGGTGTTCAAACAAACAAAAACAGTGGCGCTCGTTCGACAAATTTGATTCAGGTAGACAA
TTTGCCAAGTTCTTATATAAACACTATTAATGCTTCGGGTACTTCCCGCCCGCTTACCGTTTTGCCACCCAGTGTAACTC
AGGCTCCCACCCCCTCAGTGTGCCGTACTGTAAACATTTGCTCCGCTGTGCGCCGCTGCAGGGATAAACTTTTTGTGACT
TTGCATTTTGCTCAGCAGCCTGTTCAGTTTCAATTAGACACGGGTGCTTCTTTGTCTCTCATTAATAGTGCTACGTATGA
TTTGTTGGGTCGACCAGTTTTGTCGCCCACTGACAGACAGCTCACGTCCTACAATGGTGATCGCATTGATGTTTTAGGCA
CGTGCAGCTTACCTGCCACGTACCGCCAGTGTACTAAAACTGTCACGTTGTATGTACTGTCTTCGCCAGACTCAGTGAAT
ATTTTTGGCCTCGATCTTTTTGATTTGTTTCACATGACTATTCAAGATCGAGTGTTATCTGTGAATGCATTTCAGTCACA
TTCTGCTGTGGACAAATTGTGTGACGACTTTGCTTCTTTGTTTTCACCAGGCTTAGGTAAAGCTAAAGATTTTGAGGCTC
ACATTCAATTGCTCGATTCTGCGCAGCCTCGTTTTTTTCGCGCCCGTAAAGTTCCTCAAGCACTTCAAGAACAAGTCGCT
TTTGAACTTCGTGAATTGCAGGCACAGGGTGTGATTCAGTCCATCACTGCTAGCCAATGGGCTTCTCCCATCGTTGCTGT
GAGAAAACCGTCGGGTCGCCTACGCATTTGTGCCGACTTTAAGCCTACAGTGAACCCTCAGACTGTTATTGCTTCTTACC
CACTCCCACGTCCTGATGAATTCATGGAGCGCCTGGGAGCTGGCAAATACTTTTCGAAAATTGATTTGCGCGATGCGTAT
TTACAGTTGCCTTTGGACAATGCATCGAAATCTGTTCTTGTAATCAACACTCATTTGGGACTTTTTCGATTTTTACGTTT
GCCATTCGGGTGTGCTTCAGCTCCTGCCATTTTCCAACGGTACGTCGATCAGCTGATTGCGTCTGTTCCGTCGTGTGCTA
CTTACTTGGACGACATCATTGTTTCTGGTCGAACTCCGGAGGAACATTTGTCAAATCTCCGATGCCTTTTTCAAGTGTTA
TTATCTGCAGGACTTAAGTGTAACAAGGAGAAATGTGTTTTTTTTCAGTCGGAAATCGATTACTTGGGCCATAGGATCAA
TAGTCAGGGCATACATCCTTTGCAGGCCCACCTACTGGCAATCAAGAATCTTCCGACGCCGAAAAATTTGCAGGAACTTC
AAAGTGTCTTGGGAAAACTTACATACTACATTCGGTTCATCCCGAATGCTGCTCAAATTGCTGCGCCTTTGCACCGCTTG
CGCCGCAAAAATGTTCCTTTTGTGTGGGATAAACATTGTGATGTTGCTTTTCAGACGCTCAAAAATGAATTGCTCAGCGA
TCGTTGCCTTGTTCATTTCGATCCCTCCAGACCACTTGTGTTAGCTGCCGATGCTTCCTCATACGGCATAGGTGTTGTTC
TGTCACATAGGTTTGGGGATCACGATCGCCCCATTGCTTTTGCGTCGAAGTTGCTTACTCCCGCACAATGCAAGTACTCT
CAAATCGAGAAAGAAGCTTTAGCCCTTGTGTACGGAGTTACCAAATTTCATCATTATTTGTATGGTCGTAAGTTCTTCTT
GGTCACGGACCATAAGCCGTTGCAATCTTTGTTCCATCCGGCCAAGCCTATTCCGGATCGGACTGCTCATAAGTTGCAAC
GGTGGGCTCTTTTGTTGTCAGCCTACACTTACGAAATTTTGTATCGTCCTTCGGCGAAGCATGCGAATGCGGACGCGTTG
TCACGTCTTCCGTCCGGCCCGGACGAGTCTTTTGACGACTCGCCTGTTTCTTGTTGTGCGATGGATTCTCAGGATTCCGA
TCTTTTAGCCGCGTTTCCACTTGATTACAAACAAGTTGCGCAGGCTACTGCTTCTGACCCGGTCTTGTCTTTGTTGCTTC
GCTTTGTTCGCTCTGGTTGGCCGCGTTCCGCGCGCCACATTTCGGATTCACATGTTCGCCGTTATTTTGCTAGGCGTCAG
GATTTGTCGGTCCATGACGGTGTTCTTTTGCTCCGCACCTCCCCTGACCAGCCTCGCGTCGTTGTACCTCGTGTTCTTCA
ACGTCGTGTCTTGAATTTGTTGCATCAAGGCCATTGGGGACTCGTTCGTACAAAACAGTTAGCTCGCCGTCATTGTACGT
GGTTGCGCATGGATGCCGATATCGAGCGTCTTACCGCCAATTGTTCTGCTTGCGCCGAGCATCAAGCTGCTCCGCCGCAA
CGCTTTTTTGATTGGCCGAAATCTTCTTCGCCTTGGCAACGGTTGCACTTAGATTTCGCTGGTCCATTTTGGAACACTAG
ATGGTTGCTCTTGATCGATTCGTTCAGCGGTTTTCCTTTTGTTGTGCCGCTTTCATCGACGACAGCTGCTGCTACCATCT
CTGTTTTGCAGTCTATTTTTTGTCTGGAAGGTTTGCCTGAAACGTTGGTGACGGATAATGGACCCCAATTTGCTTCTTCT
GCTTTTGCTGATTTTTGTCGTGCCAATGGTATTATTCATCTGACCAGTGCACCGTTTCACCCTGCTTCCAATGGTGCTGC
CGAACGTCTTGTTCGCACTTTTAAGGAGCACATGTCTCGGTTGCGCTCCTCTCATTCTCGGGAGGAGGCGCTGCTGATAT
TTTTGTCCTCTTACCGCTCGCAGCCGCGTGCAGGTCCATCCCCGGCTGAACTTCTTCACGGCCGGCGACATCGCACGCTT
CTGTCTTTGCTCCGCCCACCACAGCGGGCCATGCCTCCGCCGCCGCTCCAGCCGGCCTCCCGATGGAGGTGGACTCCATC
ACGTCGTCTCCGGCTGCTTCTTAACGCGGATGGCGGGGGTGCGCCATCCGGTGGTTTTGGCCGGTTTTCCTCCGGACCGC
GCGCCGAATGGCGGGGCGCGGGCCAGCCGCTCCGGCTCCGTTCGGCGCCTGGCCTCCTCGATTGCCCCCGCCCCGCGCCC
TCCCCCCGTCGTCGCTCCTGCTACTTTCTCAGCGACGACTGGCGTCGCTTGAGGGGGGAGGGGTGCAGTATCCATGTATC
GATACTGCCGCAGCGATAGACATCGCATAGCGTCCGGCGCGAACGCTAGAGGTCGGGTCCGCGTCACCAGAGGTCGCCCC
GCGGACCGGCCGAATAGCACGCTGCCGCGCCTATATCTAGCGCGGCAGAACAGGAAGTACTCAGTCTTCCGTCGAGTCTG
CAGTGAGTCTTCAAACAAGTCTTCAACGTGAGTCTTCCGCAAGCAGTCTACAAGTTACCTTCACGCCGCGTCTACTCATC
GCTACAAGTGCTGCCTCATCTTCACTTACTGTGTATTTGTAAACCTACGACGACAGCGAGGAGGATGTACCCAAGTTACG
TAATTATTCAGTCATAATAAATCTTTCACTATTATTGACTTGTTTGCTTTCTGTTTTTGATGTGTCGAGCAAGGACACAT
CAACTGGCGACGAGGATGGGATATTCTCTCGCTGCCTTGGTTTGTCGCAATTTTTTTTTTCTGGTTAGTCAGTAAGCTAT
TTTTGCTTTCGCCGTTGCTTCATAATTTTGTTTTCATCGCTGGATGCGATTTATATGCACGATCGTGCACAGCACTTGCT
GCTATTGGCTGTTTTTGCTCTTTTTTGCTCTTCTTGCTTA
